# PROTEA, A Southern African Multicenter Congenital Heart Disease Registry and Biorepository: Rationale, Design, and Initial Results

**DOI:** 10.3389/fped.2021.763060

**Published:** 2021-10-20

**Authors:** Thomas Aldersley, John Lawrenson, Paul Human, Gasnat Shaboodien, Blanche Cupido, George Comitis, Rik De Decker, Barend Fourie, Lenise Swanson, Alexia Joachim, Phaphama Magadla, Malebogo Ngoepe, Liam Swanson, Alistair Revell, Raj Ramesar, Andre Brooks, Nicole Saacks, Bianca De Koning, Karen Sliwa, John Anthony, Ayesha Osman, Bernard Keavney, Liesl Zühlke

**Affiliations:** ^1^Division of Paediatric Cardiology, Department of Paediatrics and Child Health, University of Cape Town, Cape Town, South Africa; ^2^Division of Paediatric Cardiology, Department of Paediatrics and Child Health, University of Stellenbosch, Cape Town, South Africa; ^3^Chris Barnard Division of Cardiothoracic Surgery, University of Cape Town and Groote Schuur and Red Cross Children's Hospitals, Cape Town, South Africa; ^4^Department of Medicine, Cape Heart Institute, University of Cape Town, Cape Town, South Africa; ^5^Division of Cardiology, Department of Medicine, University of Cape Town, Cape Town, South Africa; ^6^Department of Mechanical Engineering, University of Cape Town, Cape Town, South Africa; ^7^Department of Mechanical, Aerospace and Civil Engineering, The University of Manchester, Manchester, United Kingdom; ^8^Division of Human Genetics, Department of Pathology, University of Cape Town, Cape Town, South Africa; ^9^Division of Maternal and Foetal Medicine, The Department of Obstetrics and Gynaecology, University of Cape Town, Cape Town, South Africa; ^10^Division of Cardiovascular Sciences, School of Medical Sciences, Faculty of Biology, Medicine and Health, The University of Manchester, Manchester, United Kingdom; ^11^Manchester University NHS Foundation Trust, Manchester Academic Health Science Centre, Manchester, United Kingdom

**Keywords:** congenital heart disease, Africa, epidemiology, genetics, registry, biorepository

## Abstract

**Objectives:** The PartneRships in cOngeniTal hEart disease (PROTEA) project aims to establish a densely phenotyped and genotyped Congenital Heart Disease (CHD) cohort for southern Africa. This will facilitate research into the epidemiology and genetic determinants of CHD in the region. This paper introduces the PROTEA project, characterizes its initial cohort, from the Western Cape Province of South Africa, and compares the proportion or “cohort-prevalences” of CHD-subtypes with international findings.

**Methods:** PROTEA is a prospective multicenter CHD registry and biorepository. The initial cohort was recruited from seven hospitals in the Western Cape Province of South Africa from 1 April 2017 to 31 March 2019. All patients with structural CHD were eligible for inclusion. Descriptive data for the preliminary cohort are presented. In addition, cohort-prevalences (i.e., the proportion of patients within the cohort with a specific CHD-subtype) of 26 CHD-subtypes in PROTEA's pediatric cohort were compared with the cohort-prevalences of CHD-subtypes in two global birth-prevalence studies.

**Results:** The study enrolled 1,473 participants over 2 years, median age was 1.9 (IQR 0.4–7.1) years. Predominant subtypes included ventricular septal defect (VSD) (339, 20%), atrial septal defect (ASD) (174, 11%), patent ductus arteriosus (185, 11%), atrioventricular septal defect (AVSD) (124, 7%), and tetralogy of Fallot (121, 7%). VSDs were 1.8 (95% CI, 1.6–2.0) times and ASDs 1.4 (95% CI, 1.2–1.6) times more common in global prevalence estimates than in PROTEA's pediatric cohort. AVSDs were 2.1 (95% CI, 1.7–2.5) times more common in PROTEA and pulmonary stenosis and double outlet right ventricle were also significantly more common compared to global estimates. Median maternal age at delivery was 28 (IQR 23–34) years. Eighty-two percent (347/425) of mothers used no pre-conception supplementation and 42% (105/250) used no first trimester supplements.

**Conclusions:** The cohort-prevalence of certain mild CHD subtypes is lower than for international estimates and the cohort-prevalence of certain severe subtypes is higher. PROTEA is not a prevalence study, and these inconsistencies are unlikely the result of true differences in prevalence. However, these findings may indicate under-diagnosis of mild to moderate CHD and differences in CHD management and outcomes. This reemphasizes the need for robust CHD epidemiological research in the region.

## Introduction

Congenital heart disease (CHD) is common, affecting 9 per 1,000 live births, and contributes significantly to the global burden of disease (1–3). In addition, CHD constitutes one-third of all congenital birth defects (4), a leading cause of childhood mortality (5). Thus, CHD is increasingly recognized as an important focus in the reduction of under-5 deaths and the realization of the United Nations', 2016, Sustainable Development Goals (3, 6, 7).

Accurate and contemporary epidemiological research is an essential first step in this process, unfortunately, epidemiological data from Africa and low-income countries are lacking. Recent analyses of the global prevalence of CHD show significant geographic variation in reported prevalence rates, with African prevalence rates significantly lower than in other parts of the world (4, 6, 8). The study by van der Linde et al. (4) a meta-analysis of 114 papers from 1930 to 2010, found a global CHD birth prevalence of 9.1 per 1,000 live births. African data, however, indicated a birth prevalence of only 1.9 per 1,000 live births, significantly lower than all other regions. Similarly, the findings of Liu et al. (8), a recent global meta-analysis of 260 studies from 1970 to 2017, show that the reported birth prevalence of CHD in Africa was 2.3 per 1,000 live births, significantly lower than the global prevalence of 9.4 per 1,000 live births reported in the same study.

**Figure 1:**
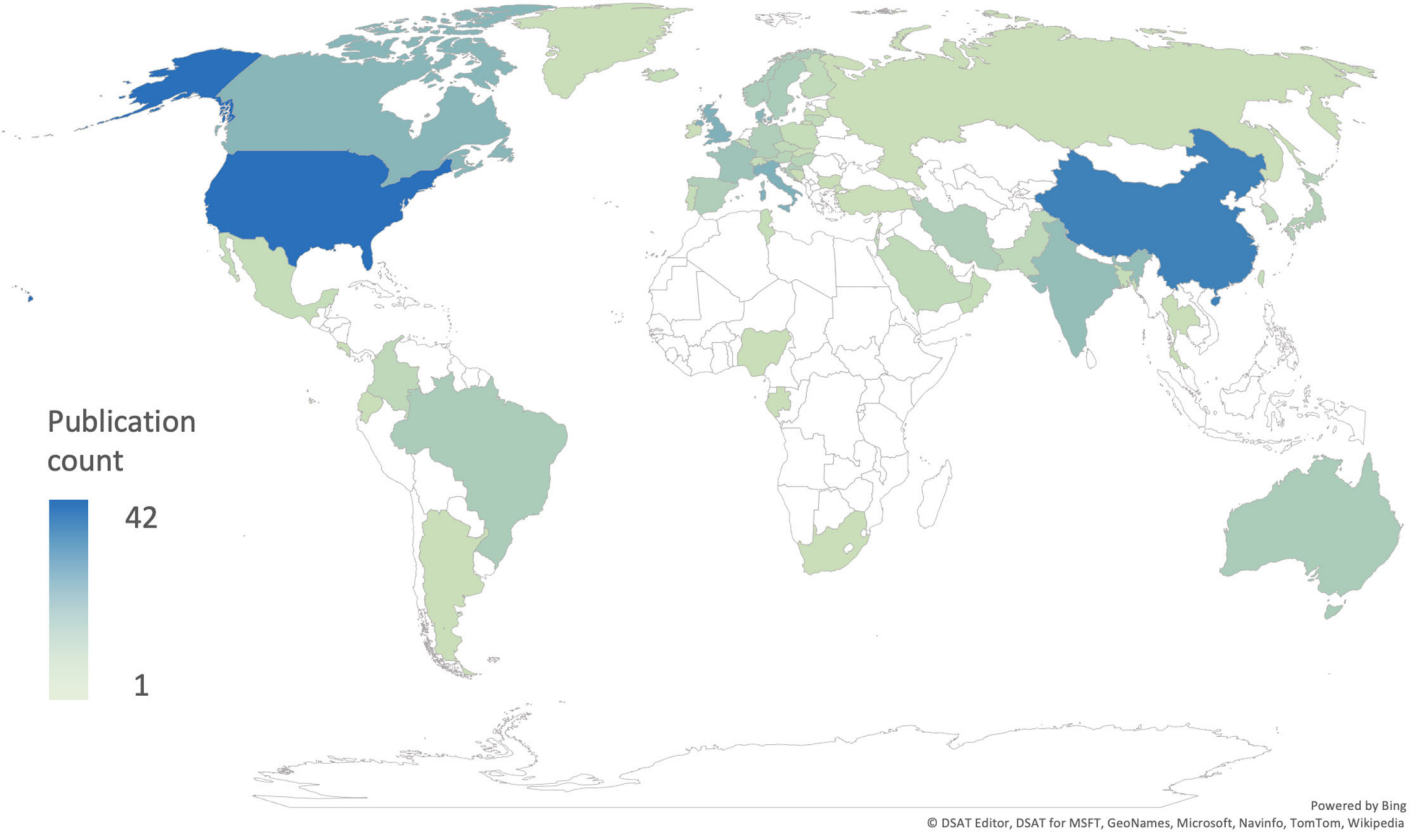
Number of publications per country included in the global prevalence studies by Liu et al. (8) and van der Linde et al. (4).

These results do not represent the true prevalence of CHD in Africa but rather reflect the extreme paucity of up-to-date research into CHD prevalence in the region (Figure 1). This premise is supported by the available literature, which documents the high burden of CHD (9–13). Rigorous, contemporary epidemiological data on sub-Saharan CHD are of immediate practical importance to inform and guide healthcare agencies and policymakers.

Similarly, the genetic architecture of the sub-Saharan CHD population is still largely understudied and the contribution of environmental factors unexplored (14). Region-specific genetic research is important for several reasons. Africa, as the birthplace of *Homo sapiens*, is home to genetically diverse populations. This genetic diversity renders them particularly powerful for identifying causative genetic variants (15). Despite this genetic diversity African populations are under-represented in DNA databases and there is a drive to correct this omission, importantly, through the development of African-hosted DNA bio-repositories (16, 17). More specifically, studies into the genetic architecture of cardiovascular disease have shown differences between Europe and sub-Saharan Africa (18), and the contribution of genetic syndromes and *de novo* mutations, in known CHD genes, is still unknown and may be clinically important.

The PROTEA (PartneRships in cOngeniTal hEart disease in Africa) project was created to determine the feasibility of maintaining a densely phenotyped and genotyped longitudinal CHD cohort in southern Africa. This cohort would facilitate future studies to address the lack of epidemiological and genetic data on CHD in southern Africa and help to develop clinical and cardiogenetic research infrastructure in the region.

The PROTEA project has four main aims. Aim 1 is to describe the phenotype and clinical management of CHD in southern Africa, following the implementation of a multicenter CHD registry and biorepository initially based in the Western Cape public cardiology service. Aim 2 is to investigate the genetic and molecular determinants of CHD in the region. Aim 3 is to study repaired tetralogy of Fallot and coarctation of the aorta using computational fluid dynamics, to demonstrate its potential to assist clinical assessment of CHD including long-term prediction of growth and remodeling from local blood flow (19). The growing pool of data from Aims 1, 2, and 3 will support development of the “digital twin” concept (20). Here, the combination of computational physics, artificial intelligence and machine learning will enable model-based patient-specific outcome assessment.

Finally, aim 4 is to build capacity for CHD research in southern Africa through the development of expertise and a sustainable research infrastructure. In addition, the PROTEA project will disseminate an integrated CHD electronic health record system (EHR) and research database. This is one of the key strengths of the project and distinguishes PROTEA from other registries. Many African centers have limited means to capture and store patient records electronically and the PROTEA application will greatly benefit their clinical practice. For example, PROTEA enables immediate access to medical reports, improved clinical audit processes, insight into mortalities and morbidities and related opportunities for learning. Additionally, PROTEA provides teaching opportunities via instructional clinical record forms and facilitates the opportunity for future research.

This paper will introduce the PROTEA project and characterize its initial cohort from the Western Cape province of South Africa, enrolled over a 2-year period from 1 April 2017 to 31 March 2019. In addition, the “cohort-prevalences” (i.e., the proportion of patients within the cohort with a specific CHD-subtype) of CHD subtypes in PROTEA's pediatric-cohort is compared with CHD subtype cohort-prevalences as described in two recent global meta-analyses of CHD birth-prevalence (4, 8).

## Methods

### Study Design

The PROTEA study is a prospective cohort of CHD in both children and adults which commenced in April 2017. The aim was to enroll 1,200 registry participants and collect 500 DNA repository samples over a 2-year period from April 1, 2017 to March 31, 2019. Enrolment is ongoing.

### Setting and Population

**Figure 2:**
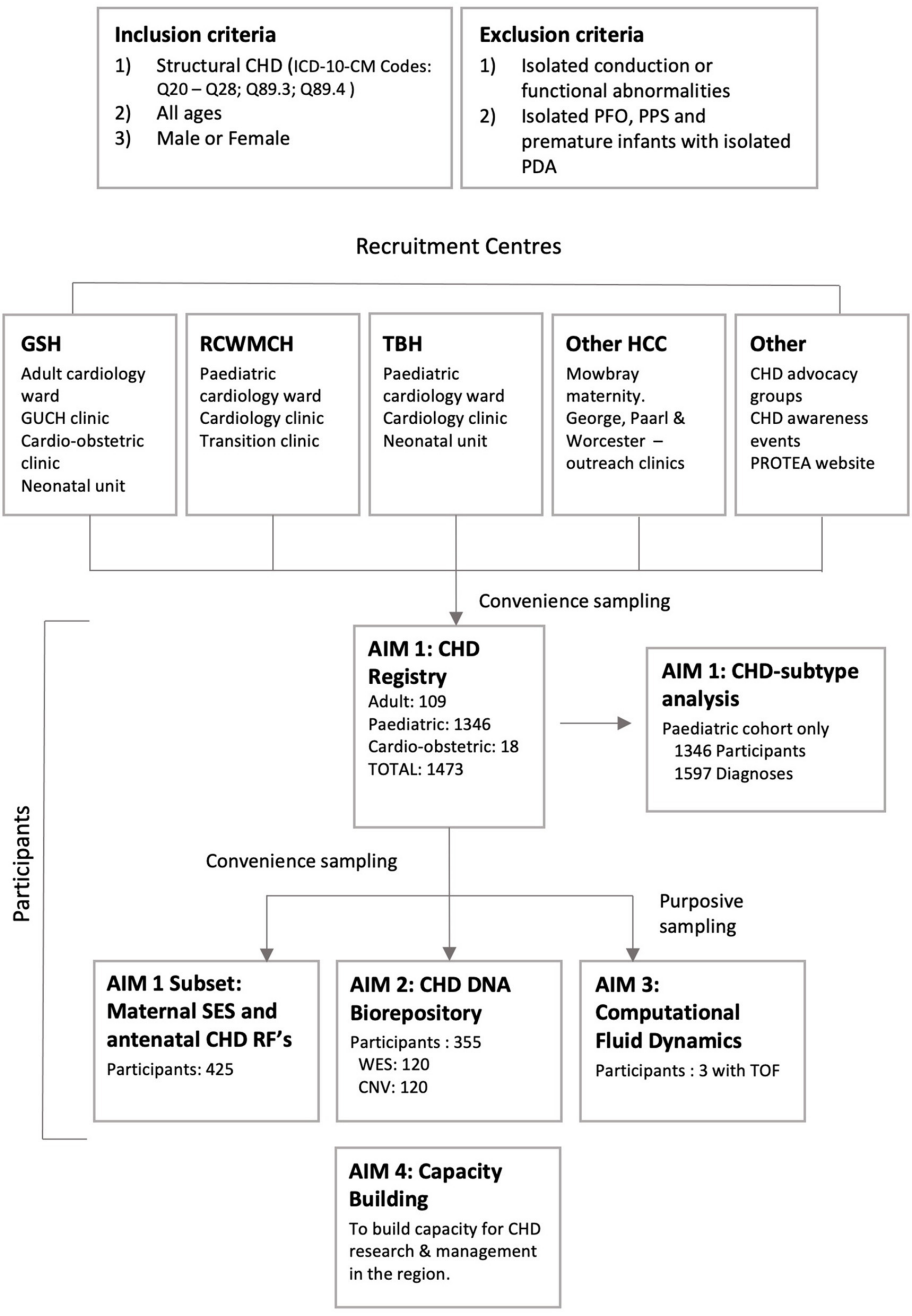
PROTEA recruitment: Inclusion and exclusion criteria, participating centers, and recruitment to aims 1–3. GSH, Groote Schuur Hospital; RCWMCH, Red Cross War Memorial Children's Hospital; TBH, Tygerberg Hospital; HCC, Health Care Centre; CNV, Copy Number Variant analysis; GUCH, Grown-Up Congenital Heart; PDA, patent ductus arteriosus; PFO, patent foramen ovale; PPS, peripheral pulmonary stenosis; RF, Risk Factor; SES, Socio-economic status; TOF, tetralogy of Fallot; WES, Whole Exome Sequencing.

Patients are recruited to Aim 1, the CHD registry, via convenience sampling primarily from three tertiary centers in the Western Cape Province of South Africa: Red Cross War Memorial Children's Hospital (RCWMCH), Tygerberg Hospital (TBH) and Groote Schuur Hospital (GSH) via the neonatal, pediatric, adult, and obstetric clinics and wards. Participants are also enrolled from the Mowbray Maternity Hospital, pediatric cardiology outreach clinics at George, Paarl, and Worcester Hospitals, and via engagement with CHD advocacy groups and CHD awareness events (Figure 2). Additionally, recruitment has begun at Windhoek Central Hospital, Namibia, however these participants are not included in this analysis. To minimize selection bias, recruitment to Aim 1 was systematic. All patients referred to the above-mentioned cardiology service were screened via folder review (for prevalent cases) and echocardiogram (for all incident and certain prevalent cases). All patients found to have structural CHD and fitting the inclusion and exclusion criteria were invited to participate in Aim 1.

Aim 2 and 3 participants are selected from Aim 1 via convenience and purposive sampling, respectively. Additionally, a convenience sample of pediatric participants admitted to the RCWMCH cardiology ward were selected for interview regarding socioeconomic status and maternal perinatal risk factors for CHD.

### Inclusion and Exclusion Criteria

All patients with an echocardiogram-confirmed diagnosis of structural CHD are considered eligible for inclusion in the study. Participants with isolated conduction or functional abnormalities, patent foramen ovale, peripheral pulmonary stenosis or patent ductus arteriosus in premature infants were excluded.

### Analysis

The proportion of CHD-subtypes in PROTEA's pediatric cohort was compared with the proportion of CHD subtypes in two global CHD birth-prevalence studies by van der Linde et al. (4) and Liu et al. (8). Twenty-six CHD-subtypes were selected for comparison. These subtypes were selected to match the ICD 9 and 10 subtype data presented in Liu et al. (8). Van der Linde et al. (4) only present data for the 8 most common CHD-subtypes in their analysis, all of which are included in the 26 subtypes above.

Cohort-prevalence ratios were calculated using R (version 4.0.0, R Foundation) (21) and the R-package epiR (version 1.0-14, Stevenson 2020) (22). Contingency tables were created for each CHD subtype and used to calculate prevalence ratios between PROTEA and both Liu et al. (8) and van der Linde et al. (4) independently. The 95% confidence intervals (CI) for the prevalence ratios were calculated using the Wald method, in addition *p*-values were generated using the chi-square test for independence, *p* < 0.05 were considered significant.

### Aims 2 and 3

The methods and results of aims 2 and 3 are beyond the scope of this paper and will be presented in future articles (19).

### Data Management and Security

All data are stored in the PROTEA application and database. The PROTEA application was developed using FileMaker (Claris International Inc., Santa Clara, CA) and integrates an EHR with a research database. Security features include encryption of data at rest, hierarchical access control and data encryption between client and server. Data integrity is ensured via intelligent prompting, audit logs recording all changes as well as incremental backups to geographically separated, redundant disk arrays.

## Results

### Enrolment

Over the initial 2-year period, 1,473 patients were enrolled (1,346 pediatric; 109 adult and 18 from the combined cardio-obstetric clinic); 355 participants were added to the DNA repository with whole exome sequencing and copy number variant analysis completed on 120 samples each (Figure 2). Analysis of the resulting data is in progress.

### Study Population

There were 752 (51%) male and 721 (49%) female participants. Median age by cohort was 1.5 years (Interquartile Range [IQR] 0.3–5.3) for the pediatric cohort, 23 years (IQR 18–35) for the adult cohort, and 27 years (IQR 26–33) for the cardio-obstetric cohort.

### Diagnosis

Most participants were diagnosed under 1 year (Adult 63/87, 72%; Pediatric 641/1108, 58%). However, antenatal detection was rare (Adult 9/87, 10%; Pediatric 81/425, 19%).

**Figure 3:**
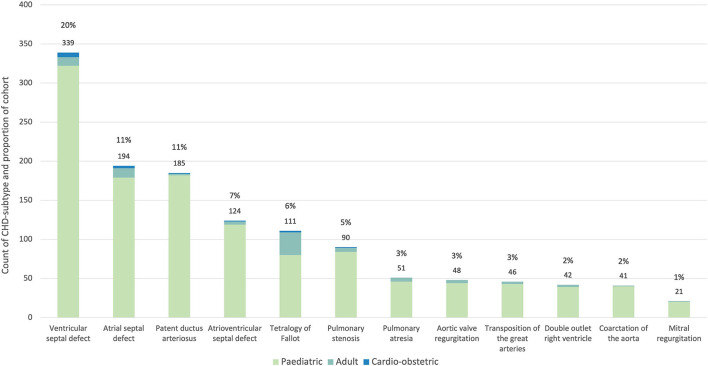
Proportion of the 12 most common CHD subtypes in the PROTEA cohort. The count and percentage values for the pooled data are given above the bars. The contribution of the pediatric (green), adult (turquoise) and cardio-obstetric (blue) cohorts is represented by the stacked bars.

Multiple diagnoses were permitted per patient with a total of 1,715 recorded diagnoses, this number included 1,597 diagnoses for the pediatric cohort, 103 for the adult cohort, and 15 for the cardio-obstetric cohort. The 12 predominant subtypes (Figure 3) were ventricular septal defect (VSD) (339, 20%), atrial septal defect (ASD) (174, 11%), patent ductus arteriosus (PDA) (185, 11%), atrioventricular septal defect (AVSD) (124, 7%), tetralogy of Fallot (TOF) (121, 7%), pulmonary stenosis (PS) (80, 5%), pulmonary atresia (PA) (48, 3%), congenital aortic regurgitation (AR) [in keeping with the ICD 9 (746.4) and 10 (Q23.1) CHD-subtype data presented in Liu et al. (8), this diagnosis includes bicuspid aortic valve] (46, 3%), transposition of the great arteries (TGA) (42, 2%), double outlet right ventricle (DORV) (41, 2%), coarctation of the aorta (CoA) (21, 1%), and congenital mitral regurgitation (MR) (21, 1%). Cohort-prevalence data are presented in full in Supplementary Table 1.

### CHD-Subtype Cohort-Prevalences

**Figure 4:**
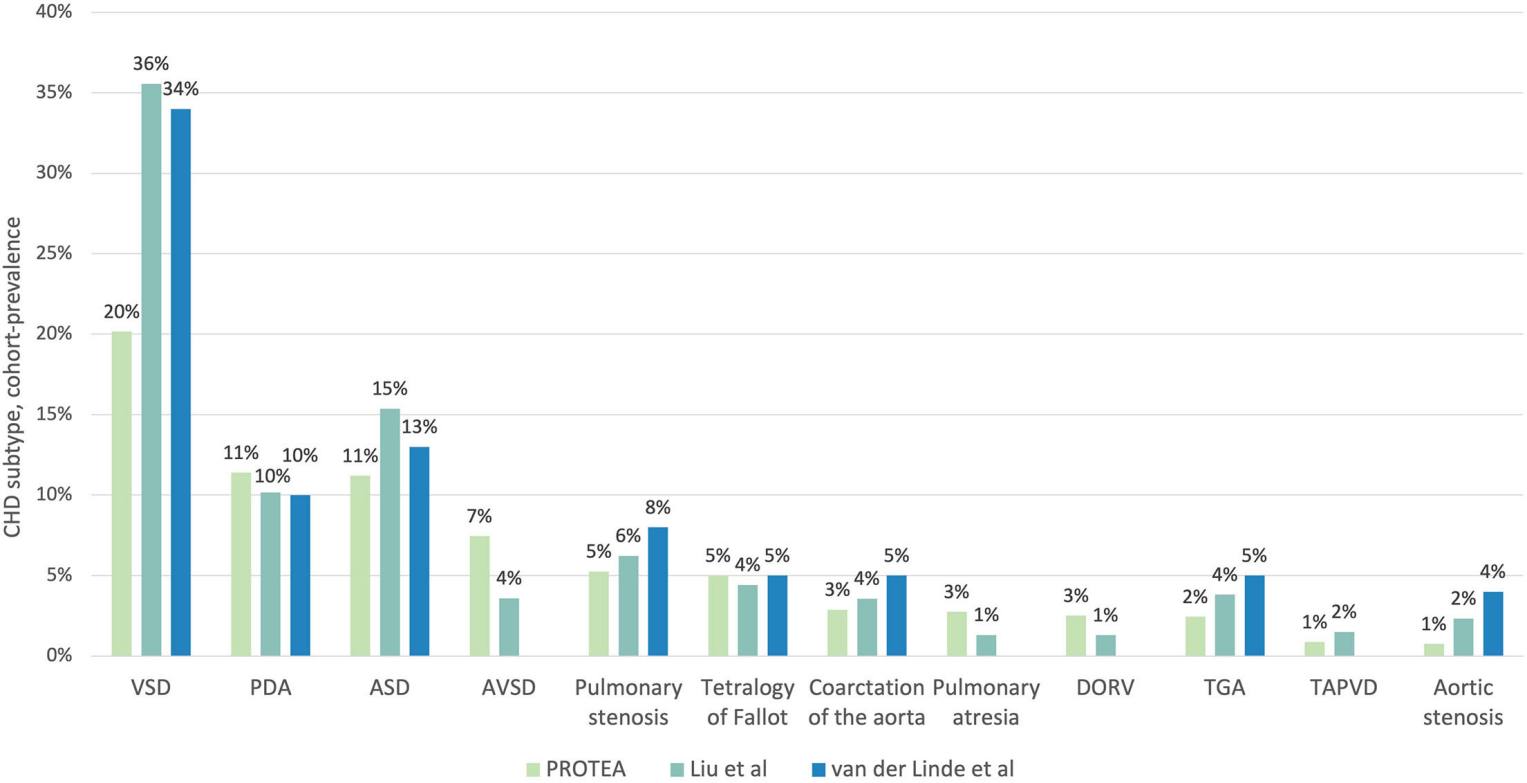
A comparison of the proportion of CHD subtypes between the PROTEA cohort (green) and the global prevalence studies by Liu et al. (8) (turquoise) and van der Linde et al. (4) (blue). Data represent the 11 most common CHD subtypes in the PROTEA cohort. DORV, double outlet right ventricle; TGA, transposition of the great arteries; TAPVD, total anomalous pulmonary venous drainage.

The prevalence of VSDs was significantly lower in the PROTEA pediatric cohort than in both Liu et al. (8) (Prevalence Ratio [PR] 0.57, 95% CI, 0.51–0.63; *p* < 0.001) and van der Linde et al. (4) (PR 0.59, 95% CI, 0.54–0.65; *p* < 0.001) (Figure 4, Table 1). Similarly, the prevalence of ASDs was significantly lower in PROTEA than in both Liu et al. (8) (PR 0.73, 95% CI, 0.63–0.834; *p* < 0.001) and van der Linde et al. (4) (PR 0.86, 95% CI, 0.75–0.99; *p* = 0.034). The prevalence of PDAs was not significantly different. The pediatric cohort-prevalences of AVSDs (PR 2.07, 95% CI, 1.74–2.46; *p* < 0.001), PA (PR 2.11, 95% CI 1.57–2.82; *p* < 0.001), and DORV (PR 1.92, 95% CI 1.1.41–2.61; *p* < 0.001) were significantly higher in PROTEA than Liu et al. (8). The prevalence of TGA was significantly lower in PROTEA than both van der Linde et al. (4) (PR 0.49, 95% CI, 0.36–0.67; *p* < 0.001) and Liu et al. (8) (PR 0.64, 95% CI, 0.47–0.87; *p* = 0.004). The prevalence of PS (PR 0.66, 95% CI, 0.53–0.81; *p* < 0.001) and CoA (PR 0.59, 95% CI, 0.43–0.77; *p* < 0.001) was significantly lower in PROTEA than van der Linde et al. (4) but not Liu et al. (8). The results of all 26 CHD subtypes are included in Supplementary Table 2.

**Table 1:** PROTEA CHD-subtype pediatric^*a*^ cohort-prevalence compared with the global prevalence studies by Liu et al. (8) and van der Linde et al. (4).

	**PROTEA**	**Liu et al**. **(****8****)**			**van der Linde et al**. **(****4****)**		
	**(*N* = 1,597)**	**(*****N*** **= 1,161,030)**			**(*****N*** **= 164,396)**		
**CHD Subtype**	**No. (%)**	**No. (%)**	**PR (95%CI)**	***p***	**No. (%)**	**PR (95%CI)**	***p***
**Ventricular septal defect (Q21.0)**	**322 (20)**	**412,955 (36)**	**0.57 (0.51:0.63)**	***p*** **< 0.001**	**55,895 (34)**	**0.59 (0.54:0.65)**	***p*** **< 0.001**
Patent ductus arteriosus (Q25.0)	182 (11)	118,100 (10)	1.12 (0.98:1.28)	*p* = 0.11	16,440 (10)	1.14 (0.99:1.31)	*p* = 0.064
**Atrial septal defect (Q21.1)**	**179 (11)**	**178,543 (15)**	**0.73 (0.63:0.84)**	***p*** **< 0.001**	**21,371 (13)**	**0.86 (0.75:0.99)**	***p*** **= 0.034**
**Atrioventricular septal defect (Q21.2)**	**119 (7)**	**41,739 (4)**	**2.07 (1.74:2.46)**	***p*** **< 0.001**	**NR (≤4)** ^b^	**-**	**-**
**Pulmonary stenosis (Q22.1)**	**84 (5)**	72,367 (6)	0.84 (0.69:1.04)	*p* = 0.11	**13,152 (8)**	**0.66 (0.53:0.81)**	***p*** **< 0.001**
Tetralogy of Fallot (Q21.3)	80 (5)	51,341 (4)	1.13 (0.91:1.4)	*p* = 0.25	8,220 (5)	1 (0.81:1.24)	*p* = 0.99
**Coarctation of the aorta (Q25.1)**	**46 (3)**	41,449 (4)	0.81 (0.61:1.07)	*p* = 0.14	**8,220 (5)**	**0.58 (0.43:0.77)**	***p*** **< 0.001**
**Pulmonary atresia (Q22.0)**	**44 (3)**	**15,186 (1)**	**2.11 (1.57:2.82)**	***p*** **< 0.001**	NR	-	-
Aortic regurgitation (Q23.1)	43 (3)	26,913 (2)	1.16 (0.86:1.56)	*p* = 0.32	NR	-	-
**Double outlet right ventricle (Q20.2)**	**40 (3)**	**15,128 (1)**	**1.92 (1.41:2.61)**	***p*** **< 0.001**	NR	-	-
**Transposition of the great arteries (Q20.3)**	**39 (2)**	**44,340 (4)**	**0.64 (0.47:0.87)**	***p*** **= 0.004**	**8,220 (5)**	**0.49 (0.36:0.67)**	***p*** **< 0.001**
Tricuspid atresia or stenosis (Q22.4)	17 (1)	12,435 (1)	0.99 (0.62:1.6)	*p* = 0.98	NR	-	-
**TAPVR (Q26.2)**	**14 (1)**	**17,427 (2)**	**0.58 (0.35:0.98)**	***p*** **= 0.04**	NR	-	-
**Aortic stenosis (Q23.0)**	**12 (1)**	**27,098 (2)**	**0.32 (0.18:0.57)**	***p*** **< 0.001**	**6,576 (4)**	**0.19 (0.11:0.33)**	***p*** **< 0.001**

*Significant differences between CHD subtype cohort-prevalence are highlighted in bold and in pink where the PROTEA cohort-prevalence is lower or in green where it is higher. See Supplementary Table 2 for all results*.

a *The data represents the PROTEA pediatric cohort only*.

b *Atrioventricular septal defect data is not presented in van der Linde et al. (4), results are inferred from lowest presented proportion (Aortic Stenosis 4%). PR, prevalence ratio; NR, Not reported; TAPVR, Total anomalous pulmonary venous return*.

### Maternal Cohort

In mothers of pediatric CHD participants median age at index delivery was 28 years (IQR 23–34). Only 153/425 (36%) were employed with 99/153 (65%) earning ≤ ZAR 5,000/month (±USD 300/month). During their index pregnancy, 40/425 (9%) mothers received antiretroviral therapy (ART), 26/425 (6%) had diabetes mellitus, 6/425 (1.4%) used treatment for psychiatric illness, 4/425 (0.9%) had epilepsy, 1/425 (0.2%) had thyroid disease, and 1/425 (0.2%) had systemic lupus erythematosus. Most (82%, 347/425) used no pre-conception supplementation (including folic acid) and 42% (105/250) used no first trimester supplementation.

## Discussion

This first report of the PROTEA cohort revealed proportions of CHD subtypes that were significantly different from global estimates (4, 8). The cohort-prevalence of VSDs and ASDs was as much as 1.8 (95% CI, 1.6–2.0; *p* < 0.001) and 1.4 (95% CI, 1.2–1.6; *p* < 0.001) times higher for international estimates than in the PROTEA pediatric cohort, respectively. Similarly, the cohort-prevalences of PS and CoA were significantly higher in van der Linde et al. (4) than in PROTEA and the prevalence of AS was higher for both Liu et al. (8) and van der Linde et al. (4). In contrast, AVSDs were 2.1 (95% CI, 1.7–2.5; *p* < 0.001) times more common in the PROTEA pediatric cohort than in Liu et al. (8) (Table 1). The proportion of AVSDs is not reported in van der Linde et al. (4) as AVSDs did not fall within the 8 most prevalent subtypes. It can be assumed, however, that the proportion was ≤4%, the lowest reported proportion, and thus significantly lower than the PROTEA cohort. Similarly, the cohort-prevalences of PA, and DORV were significantly lower in Liu et al. (8) than in PROTEA. In contrast, the prevalence of TGA was higher for both Liu et al. (8) and van der Linde et al. (4) than for PROTEA and the prevalence of PDAs was not significantly different.

Globally the prevalence of CHD is increasing, largely due to the increased availability and technical capability of echocardiography (8) which has resulted in increased diagnosis and reported prevalence of mild lesions like ASDs, PDAs, and VSDs. In fact, ASDs, PDAs and VSDs combined, accounted for 93.4% of the increased overall prevalence of CHD from 1970 to 2017, as reported in Liu et al. (8). The prevalence of severe CHD subtypes has remained relatively constant but with a decrease in prevalence of left ventricular outflow tract obstructions, conotruncal defects and AVSDs, likely the result of improved antenatal ultrasonography and the elective termination of affected pregnancies (TOP) (8, 23). Globally these trends have resulted in an increased proportion of mild CHD subtypes and a reduction in the proportion of severe CHD subtypes.

South Africa's reported prevalence rates may not follow this trend (9). Despite being listed as an upper middle income nation by the World Bank, South Africa is a dual economy with a high degree of income inequality (24) and associated inequalities in health care access (25). There is no official South African, Department of Health policy regarding newborn screening for critical CHD (26) and neither cardiac examination nor chest auscultation are prescribed for well-child visits or in the management of sick children at primary health care centers (27–30). As a result, it is likely that many South African children with mild CHD remain undiagnosed and this may be reflected by the lower proportions of VSDs, ASDs, AS, CoA, and PS seen in the PROTEA cohort.

Internationally, the proportion of severe CHD is decreasing, primarily due to the increase in mild subtypes but possibly also the result of increased antenatal detection of severe CHD and elective TOP (8). Indeed, Liu et al. (8) reported a decrease by approximately one-third in the estimated prevalence of left ventricular outflow tract obstruction from 0.689 (95% CI, 0.607–0.776) per thousand in 1995–99 to 0.475 (95% CI, 0.392–0.565) per thousand in 2010–17 (*p* = 0.023 for the decreasing trend, 1995–2017). In contrast, the proportion of severe CHD subtypes in the PROTEA cohort remains high. This is likely a consequence of the lower proportion of mild CHD subtypes in the cohort but increased detection and referral rates for severe CHD-subtypes relative to mild and moderate subtypes may have contributed to this finding. AVSDs, PA and DORV are associated with early and severe symptoms which are less likely to be missed during routine examination. AVSDs, in particular, are associated with trisomy-21 and infants with this well-recognized syndrome are routinely referred for full cardiac workup even when asymptomatic. In addition, poor adherence to antenatal prevention strategies, limited access to antenatal ultrasound and reduced antenatal diagnosis of severe CHD, in combination with physical, cultural and religious barriers that reduce access to TOP services (26, 31) may have contributed to higher proportions of severe CHD in the PROTEA cohort. Without true prevalence data, inferences in this regard are speculative, however our findings show low antenatal detection rates (Adult 10%, Pediatric 19%) and low rates of antenatal folate supplementation which may support this hypothesis.

Interestingly, PROTEA's findings are similar to other African, CHD cohorts and registries (10–12) which show lower proportions of VSDs (16–27%) and ASDs (6–12%) and higher proportions of AVSDs (6–9%), DORV (3%) and TOF (7–17%) consistently across all cohorts (Table 2). These similarities may be the result of related sampling strategies and their inherent biases however we think it more likely that they reflect similarities in the health care landscape, including diagnosis & reporting rates, management, and early mortality rates.

**Table 2:** CHD-subtype cohort-prevalences across 4 African studies (10–12) compared with the global birth prevalence meta-analysis by Liu et al. (8).

**Study**	**Sulafa and Karani (12)**,	**Ekure et al. (11)**,	**PROTEA**,	**Namuyonga et al. (10)**,	**Liu et al. (8)**,
	**Sudan**	**Nigeria**	**South Africa**	**Uganda**	**Global**
	***N* = 435**	***N* = 1,296**	***N* = 1,597**	***N* = 3,526**	***N* = 116,1030**
**CHD-Subtype**	**Cohort-prevalence %**				
**Ventricular septal defect (Q21.0)**	**16**	**25**	**20**	**27**	36
Patent ductus arteriosus (Q25.0)	5	12	11	22	10
**Atrial septal defect (Q21.1)**	**6**	**12**	**11**	**9**	15
**Atrioventricular septal defect (Q21.2)**	**9**	**6**	**7**	**8**	4
Pulmonary stenosis (Q22.1)	6	2	5	6	6
**Tetralogy of Fallot (Q21.3)**	**18**	**12**	**5**	**7**	4
**Coarctation of the aorta (Q25.1)**	**0.7**	**0.8**	**3**	**0.4**	4
Pulmonary atresia (Q22.0)	3	0.4	3	2.0	1
Aortic regurgitation (Q23.1)	Not reported	0.4	3	0.2	2
**Double outlet right ventricle (Q20.2)**	**3**	**3**	**3**	**3**	1
Transposition of the great arteries (Q20.3)	7	2	2	2	4
Tricuspid atresia or stenosis (Q22.4)	3	2	1	2	1
Total anomalous pulmonary venous return (Q26.2)	1	0.5	1	0.2	1
Aortic stenosis (Q23.0)	1	Not reported	1	0.9	2

*Results are highlighted in bold and in pink where African cohort-prevalences are lower or in green where they are higher*.

## Limitations

The PROTEA cohort is a convenience sample of patients with CHD presenting to the Western Cape CHD service, as such the external validity of the PROTEA cohort is at risk due to potential sampling bias. In addition, like all hospital-based registries, the true size of PROTEA's source population is technically unknown. This is due to factors such as, ill-defined referral areas and differences in availability and accessibility of health services within the source population. Accordingly, the data may not be generalizable and should not be used to make inferences about the true population prevalence of CHD or CHD subtypes in the region. However, one can use the proportion of CHD subtypes within the cohort, the “cohort-prevalence” to make comparisons with findings in other studies as, in this case the denominator, the total number of confirmed CHD cases, is known. Importantly, differences in “cohort-prevalences” of CHD-subtypes may result from differences in sampling strategy, inclusion and exclusion criteria or diagnosis classification. However, as we believe to be the case here, they may indicate differences in diagnosis and reporting rates, management, and outcomes in CHD-subtypes in the region and need investigating.

## Conclusion

The comparison of PROTEA's pediatric CHD cohort with international prevalence studies shows interesting differences in the proportions of CHD-subtypes, and these differences warrant further investigation. The lower proportion of mild CHD may indicate missed diagnoses that untreated could lead to unnecessary morbidity and mortality. The higher proportion of severe subtypes is likely a consequence of the lower proportion of mild CHD-subtypes but increased detection and reporting rates, relative to mild subtypes, may contribute to this trend. Additionally, poor primary prevention, reduced antenatal detection and lower TOP rates may have resulted in South Africa not experiencing the same degree of reduction in prevalence of certain severe subtypes that has been seen internationally. Certainty in this regard, is essential to guide prevention strategies, antenatal and post-natal screening practices, and the allocation of resources in the management of CHD. Importantly, these findings highlight the urgent need for robust epidemiological research into CHD in the southern African region, including a thorough and accurate CHD birth prevalence study.

## Patient and Public Involvement

The design and implementation of the PROTEA project was governed by a steering committee whose members include CHD patients, parents, and advocacy group leaders. The PROTEA research group hosts annual CHD awareness events for patients and families. The focus of these events is to educate on CHD, give feedback on current research and to discuss future research goals.

## Data Availability Statement

The raw data supporting the conclusions of this article will be made available by the authors, without undue reservation.

## Ethics Statement

The studies involving human participants were reviewed and approved by the University of Cape Town, Faculty of Health Sciences, Human Research Ethics Committee (R017-2014). Written informed consent to participate in this study was provided by the participants or by their legal guardian/next of kin, where appropriate.

## Author Contributions

TA, GC, BC, RD, BF, PH, AJ, JL, PM, LeS, and LZ: acquisition, analysis, and interpretation of data. TA, PH, BK, JL, AR, GS, and LZ: drafting of manuscript. All authors: conceptualization and critical revision of manuscript.

## Funding

The PROTEA project was funded by the UK Research and Innovation (UKRI) Global Challenges Research Fund (MR/P025463/1), with co-funding from the Universities of Cape Town and Manchester via the UKRI DGEMBE project (ES/N01393X/1). LZ was funded by the UK Medical Research Council (MRC) and the UK Department for International Development (DFID) under the MRC/DFID Concordat agreement, via the African Research Leader Award (MR/S005242/1). BK was funded by a British Heart Foundation personal chair. BC was funded by the Women as One - Escalator Award. GS was funded by the National Research Foundation (95627, 105923) and the Medical Research Council of South Africa (416006).

## Conflict of Interest

The authors declare that the research was conducted in the absence of any commercial or financial relationships that could be construed as a potential conflict of interest.

## Publisher's Note

All claims expressed in this article are solely those of the authors and do not necessarily represent those of their affiliated organizations, or those of the publisher, the editors and the reviewers. Any product that may be evaluated in this article, or claim that may be made by its manufacturer, is not guaranteed or endorsed by the publisher.
