# Supplementary material for: PROTEA, A Southern African Multicenter Congenital Heart Disease Registry and Biorepository: Rationale, Design, and Initial Results
Source: Front Pediatr. 2021 Oct 20;9:763060. doi: 10.3389/fped.2021.763060 (PMC8564377; doi:10.3389/fped.2021.763060)
Supplement: Supplementary file 1 [file Table_1.docx]

**SUPPLEMENTARY TABLE S1: PROTEA Western Cape Cohort, CHD-subtype cohort-prevalences, complete.**

| **CHD Sub-type** | **Count** |
| --- | --- |
| Ventricular septal defect (Q21.0) | 339 |
| Atrial septal defect (Q21.1) | 194 |
| Patent ductus arteriosus (Q25.0) | 185 |
| Atrioventricular septal defect (Q21.2) | 124 |
| Tetralogy of Fallot (Q21.3) | 111 |
| Congenital pulmonary valve stenosis (Q22.1) | 90 |
| Coarctation of aorta (Q25.1) | 51 |
| Pulmonary valve atresia (Q22.0) | 48 |
| Congenital insufficiency of aortic valve (Q23.1) | 46 |
| Other congenital malformations of pulmonary artery (Q25.7) | 43 |
| Discordant ventriculoarterial connection (Q20.3) | 42 |
| Double outlet right ventricle (Q20.1) | 41 |
| Other specified congenital malformations of heart (Q24.8) | 33 |
| Congenital subaortic stenosis (Q24.4) | 32 |
| Other congenital malformations of aorta (Q25.4) | 26 |
| Congenital malformation of heart, unspecified (Q24.9) | 24 |
| Congenital mitral insufficiency (Q23.3) | 21 |
| Congenital tricuspid stenosis (Q22.4) | 21 |
| Other congenital malformations of aortic and mitral valves (Q23.8) | 19 |
| Persistent left superior vena cava (Q26.1) | 17 |
| Stenosis of aorta (Q25.3) | 16 |
| Malformation of coronary vessels (Q24.5) | 15 |
| Total anomalous pulmonary venous connection (Q26.2) | 14 |
| Common arterial trunk (Q20.0) | 13 |
| Congenital stenosis of aortic valve (Q23.0) | 13 |
| Discordant atrioventricular connection (Q20.5) | 13 |
| Stenosis of pulmonary artery (Q25.6) | 11 |
| Other congenital malformations of cardiac chambers and connections (Q20.8) | 9 |
| Dextrocardia (Q24.0) | 9 |
| Congenital mitral stenosis (Q23.2) | 9 |
| Congenital malformation of aortic and mitral valves, unspecified (Q23.9) | 8 |
| Congenital malformation of cardiac chambers and connections, unspecified (Q20.9) | 8 |
| Double inlet ventricle (Q20.4) | 7 |
| Ebstein anomaly (Q22.5) | 7 |
| Partial anomalous pulmonary venous connection (Q26.3) | 6 |
| Other congenital malformations of great veins (Q26.8) | 6 |
| Isomerism of atrial appendages (Q20.6) | 6 |
| Other congenital malformations of tricuspid valve (Q22.8) | 6 |
| Congenital malformation of cardiac septum, unspecified (Q21.9) | 5 |
| Pulmonary infundibular stenosis (Q24.3) | 4 |
| Cor triatriatum (Q24.2) | 3 |
| Other congenital malformations of pulmonary valve (Q22.3) | 3 |
| Congenital malformation of tricuspid valve, unspecified (Q22.9) | 3 |
| Situs Inversus (Q89.3) | 3 |
| Peripheral arteriovenous malformation (Q27.3) | 2 |
| Congenital malformation of great vein, unspecified (Q26.9) | 2 |
| Other specified congenital malformations of peripheral vascular system (Q27.8) | 2 |
| Hypoplastic left heart syndrome (Q23.4) | 2 |
| Double outlet left ventricle (Q20.2) | 1 |
| Aortopulmonary septal defect (Q21.4) | 1 |
| Congenital pulmonary valve insufficiency (Q22.2) | 1 |
| Total | 1715 |
