# Supplementary material for: PROTEA, A Southern African Multicenter Congenital Heart Disease Registry and Biorepository: Rationale, Design, and Initial Results
Source: Front Pediatr. 2021 Oct 20;9:763060. doi: 10.3389/fped.2021.763060 (PMC8564377; doi:10.3389/fped.2021.763060)
Supplement: Supplementary file 2 [file Table_2.docx]

**SUPPLEMENTARY TABLE S2: PROTEA CHD-subtype pediatric^a^ cohort-prevalence compared with the global prevalence studies by Liu et al (8) and van der Linde et al (4), Complete.**

|  | **PROTEA (N =1,597)** | **Liu et al**  **(N = 1,161,030)** | |  | **van der Linde et al**  **(N = 164,396)** | | |
| --- | --- | --- | --- | --- | --- | --- | --- |
| **CHD Subtype** | **No. (%)** | **No. (%)** | **PR (95%CI)** | **p** | **No. (%)** | **PR (95%CI)** | **p** |
| Ventricular septal defect (Q21.0) | 322 (20) | 412955 (36) | 0.57 (0.51:0.63) | p < 0.001 | 55895 (34) | 0.59 (0.54:0.65) | p < 0.001 |
| Patent ductus arteriosus (Q25.0) | 182 (11) | 118100 (10) | 1.12 (0.98:1.28) | p = 0.11 | 16440 (10) | 1.14 (0.99:1.31) | p = 0.064 |
| Atrial septal defect (Q21.1) | 179 (11) | 178543 (15) | 0.73 (0.63:0.84) | p < 0.001 | 21371 (13) | 0.86 (0.75:0.99) | p = 0.034 |
| Atrioventricular septal defect (Q21.2) | 119 (7) | 41739 (4) | 2.07 (1.74:2.46) | p < 0.001 | NR (≤4)^b^ | - | - |
| Pulmonary stenosis (Q22.1) | 84 (5) | 72367 (6) | 0.84 (0.69:1.04) | p = 0.11 | 13152 (8) | 0.66 (0.53:0.81) | p < 0.001 |
| Tetralogy of Fallot (Q21.3) | 80 (5) | 51341 (4) | 1.13 (0.91:1.4) | p = 0.25 | 8220 (5) | 1 (0.81:1.24) | p = 0.99 |
| Coarctation of the aorta (Q25.1) | 46 (3) | 41449 (4) | 0.81 (0.61:1.07) | p = 0.14 | 8220 (5) | 0.58 (0.43:0.77) | p < 0.001 |
| Pulmonary atresia (Q22.0) | 44 (3) | 15186 (1) | 2.11 (1.57:2.82) | p < 0.001 | - | - | - |
| Aortic regurgitation (Q23.1) | 43 (3) | 26913 (2) | 1.16 (0.86:1.56) | p = 0.32 | - | - | - |
| Double outlet right ventricle (Q20.2) | 40 (3) | 15128 (1) | 1.92 (1.41:2.61) | p < 0.001 | - | - | - |
| Transposition of the great arteries (Q20.3) | 39 (2) | 44340 (4) | 0.64 (0.47:0.87) | p = 0.004 | 8220 (5) | 0.49 (0.36:0.67) | p < 0.001 |
| Mitral insufficiency (Q23.3) | 20 (1) | 15651 (1) | 0.93 (0.6:1.44) | p = 0.74 | - | - | - |
| Tricuspid atresia or stenosis (Q22.4) | 17 (1) | 12435 (1) | 0.99 (0.62:1.6) | p = 0.98 | - | - | - |
| TAPVR (Q26.2) | 14 (1) | 17427 (2) | 0.58 (0.35:0.98) | p = 0.04 | - | - | - |
| Aortic stenosis (Q23.0) | 12 (1) | 27098 (2) | 0.32 (0.18:0.57) | p < 0.001 | 6576 (4) | 0.19 (0.11:0.33) | p < 0.001 |
| Truncus arteriosus (Q20.0) | 12 (1) | 11401 (1) | 0.77 (0.44:1.34) | p = 0.35 | - | - | - |
| Dextrocardia (Q24.0) | 8 (1) | 11924 (1) | 0.49 (0.24:0.97) | p = 0.037 | - | - | - |
| Mitral stenosis (Q23.2) | 7 (0) | 11088 (1) | 0.46 (0.22:0.96) | p = 0.034 | - | - | - |
| Single ventricle (DILV) (Q20.4) | 6 (0) | 13294 (1) | 0.33 (0.15:0.73) | p = 0.004 | - | - | - |
| PAPVR (Q26.3) | 6 (0) | 3646 (0) | 1.2 (0.54:2.66) | p = 0.66 | - | - | - |
| Interrupted aortic arch (Q25.21) | 5 (0) | 7071 (1) | 0.51 (0.21:1.23) | p = 0.13 | - | - | - |
| Cor triatriatum (Q24.2) | 3 (0) | 2845 (0) | 0.77 (0.25:2.38) | p = 0.64 | - | - | - |
| Ebstein anomaly (Q22.5) | 2 (0) | 6200 (1) | 0.23 (0.06:0.94) | p = 0.025 | - | - | - |
| Hypoplastic left heart syndrome (Q23.4) | 2 (0) | 29769 (3) | 0.05 (0.01:0.2) | p < 0.001 | - | - | - |
| Pulmonary arteriovenous aneurysm (Q25.72) | 1 (0) | 34541 (3) | 0.02 (0:0.15) | p < 0.001 | - | - | - |
| Coronary artery aneurysm (Q24.5*) | 0 (0) | 4841 (0) | - | - | - | - | - |
| Significant differences between CHD-subtype cohort-prevalence are highlighted in pink where the PROTEA cohort proportion is lower and in green where it is higher. ^a^Data represents the PROTEA pediatric cohort only. ^b^ Data not presented in van der Linde et al, inferred from lowest presented proportion (Aortic Stenosis 4%). TAPVR = total anomalous pulmonary venous return, PAPVR = partial anomalous pulmonary venous return, PR = prevalence ratio; NR = Not reported | | | | | | | |
